# Radiographically detectable intra-articular mineralization: Predictor of knee osteoarthritis outcomes or only an indicator of aging? A brief report from the osteoarthritis initiative

**DOI:** 10.1016/j.ocarto.2023.100348

**Published:** 2023-02-23

**Authors:** Hamza Ahmed Ibad, Robert M. Kwee, Elena Ghotbi, Frank W. Roemer, Ali Guermazi, Shadpour Demehri

**Affiliations:** a The Russell H. Morgan Department of Radiology and Radiological Science, Johns Hopkins University School of Medicine, Baltimore, MD, USA; b Department of Radiology, Zuyderland Medical Center, Heerlen/Sittard/Geleen, the Netherlands; c Department of Radiology, VA Boston Healthcare System, Boston University School of Medicine, Boston, MA, USA; d Department of Radiology, University of Erlangen-Nuremberg, Erlangen, Germany

**Keywords:** BMI, Body Mass Index, CT, Computed Tomography, IAM, Intra-articular mineralization, JSN, Joint Space Narrowing, MRI, Magnetic Resonance Imaging, OA, Osteoarthritis, OAI, Osteoarthritis Initiative, OARSI, Osteoarthritis Research Society International, PASE, Physical Activity Scale for the Elderly, WOMAC, Western Ontario and McMaster universities osteoarthritis index

## Abstract

**Objective:**

To determine the association between Intra-articular mineralization (IAM) and knee osteoarthritis (OA) outcomes stratified according to participants’ age.

**Methods:**

Participants from the Osteoarthritis Initiative (OAI) with baseline radiographic OA (i.e., Kellgren-Lawrence grade ≥2 with Osteoarthritis Research Society International (OARSI) atlas joint space narrowing (JSN)) in either knee were identified. Both knees and dominant hand baseline radiographs were evaluated for the presence of IAM. Whole-grade OARSI-JSN radiographic progression and increased Western Ontario and McMaster universities osteoarthritis index scores of the knees with baseline radiographic OA (assessed annually) were defined as radiographic and symptomatic progression, respectively. Cox proportional-hazards and longitudinal multilevel regression models investigated radiographic and symptomatic progression, respectively.

**Results:**

2010 participants with baseline radiographic OA in either one or both knees (N ​= ​2976) were identified. 178 participants had baseline IAM (hand radiographs ​= ​46, knee radiographs ​= ​166, both ​= ​34). An adjusted logistic regression model suggests an association between age and IAM (Odds Ratio: 1.06, 95% Confidence Interval (CI): 1.04–1.08). Presence of any IAM was not associated with whole-grade OARSI-JSN (Hazard Ratio (HR): 1.00, 95% CI: 0.73–1.37) or symptomatic progression (Estimated difference: 1.24, p-value: 0.13) in all participants. Using stratification analysis, in younger participants <60 years old, presence of any IAM was associated with radiographic progression (HR: 1.90, 95% CI: 1.01–3.60).

**Conclusion:**

Although the presence of any radiographic IAM increases with higher age and does not predict knee OA outcomes across the entire sample of OAI participants, it is associated with knee OA radiographic progression in participants aged <60.

## Summary Statement

The presence of any radiographic Intra-articular mineralization (IAM) (triangular fibrocartilage joint complex in the hand radiograph or tibiofemoral joint in the knee radiograph) is associated with aging but not an increased risk of radiographic or symptomatic knee osteoarthritis (OA) progression in all participants with baseline radiographic knee OA.

• Overall, the presence of any IAM in the baseline hand or knee radiographs was only weakly associated with higher participants' age (Odds Ratio: 1.06, 95% Confidence Interval (CI): 1.04–1.08).
• IAM (on either hand or knee radiographs) (Hazard Ratio (HR): 1.00, 95% CI: 0.73–1.37), and knee IAM (HR: 0.94, 95% CI: 0.67–1.31) were not associated with an increased risk of radiographic knee OA progression in all selected participants enrolled in OAI (age range: 45–79).
• Using stratification analysis, in younger participants <60 years old, there was an association between any IAM and radiographic knee OA progression (HR: 1.90, 95% CI: 1.01–3.60) however, such an association was not found between knee IAM and radiographic knee OA progression (HR: 1.72, 95% CI: 0.89–3.34).

## 1 Introduction

Intra-articular mineralization (IAM), defined as the presence of calcification within hyaline cartilage, fibrocartilage, menisci, or joint capsules, is commonly diagnosed on hand or knee plain radiographs [1]. Radiologically detectable IAM prevalence is estimated to be >10% and is more frequently observed in older ages [2,3]. It is most commonly indicative of calcium phosphate and calcium pyrophosphate dehydrate crystals deposition [3].

Previous works suggest an association between IAM and knee osteoarthritis (OA) [4]. Some studies suggest that IAM may contribute to OA progression while others were not able to confirm such an association [1,3,5]. Mechanisms proposed for IAM's role in OA progression include chondrocyte apoptosis induction and synovial inflammation [6]. Conversely, it is proposed that calcium-containing crystals induced chondrocyte hypertrophy/metaplasia may be a dysregulated but preservative process [1].

Soft-tissue calcifications during physiologic aging involves various non-articular cartilaginous [7] (e.g., costochondral junction), vascular [8] (e.g., phleboliths and medial arteriosclerosis), and intracranial [9,10] (e.g., pineal gland, and basal ganglia) structures. However, these findings may be pathological in younger individuals [7,9,10]. Akin to these soft-tissue calcifications, we hypothesize that IAM may contribute to OA-related outcomes, specifically radiographic/symptomatic progression, in younger individuals.

Though recent works have shown improved localization of IAM with computed tomography (CT) [11,12], radiography remains the most prevalent modality for OA and IAM assessment in practice [11]. Therefore, we investigated radiographically detectable IAM to test our hypothesis.

We aimed to determine the relationship between radiographic IAM and risk of radiographic/symptomatic knee OA progression in all participants with baseline radiographic knee OA and also by age stratification (≥60 vs. <60).

## 2 Participant Selection and methodology

### 2.1 Study design

Overall, participants aged 45–79 of all ethnicities from publicly available OAI database were included. Full OAI protocol details are available at https://nda.nih.gov/oai/study-details. Relevant exclusion criteria include individuals who have undergone/plan to undergo bilateral total knee replacements, have baseline inflammatory arthritis, or are unlikely to demonstrate joint space loss.

Of 4796 OAI participants, 2103 participants with radiographic knee OA at baseline were included. Radiographic knee OA was defined as a Kellgren-Lawrence grade ≥2 with joint space narrowing (JSN) as defined by the Osteoarthritis Research Society International (OARSI) atlas grading system for JSN and assessed for inclusion in each knee in identified participants. Ninety-three participants with unavailable IAM data were excluded. Hence, we assessed 2010 participants with radiographic OA in 2976 knees at enrollment visits. We analyzed risk of longitudinal whole-grade or more OARSI-JSN progression over a follow up period of 8-years and the estimated effect of IAM on Western Ontario and McMaster universities osteoarthritis index (WOMAC) total scores between enrollment and last available follow-up visits.

### 2.2 Knee radiographs and IAM

**Fig. 1:**
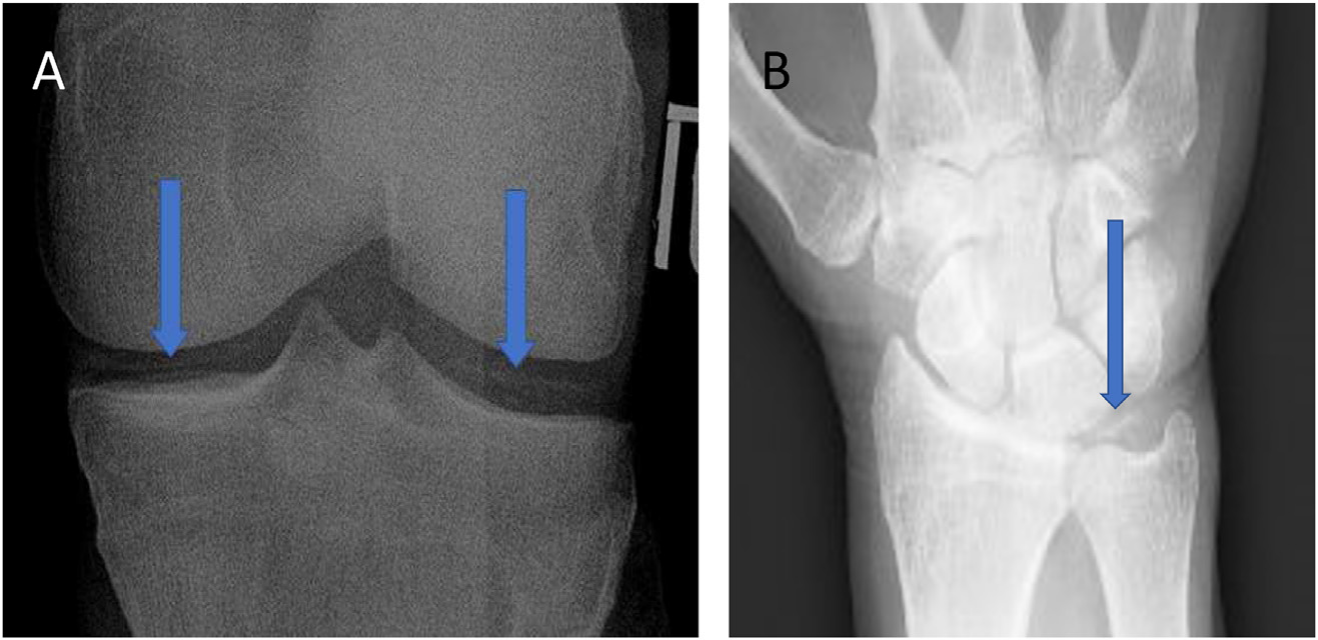
(A) shows medial and lateral IAM in the Tibiofemoral Joint. (B) depicts IAM in the Triangular Fibrocartilage Cartilage Complex.

Each knee with baseline radiographic OA was assessed for the presence of IAM. In the OAI database, radiographic knee IAM is characterized by the presence of definite linear cartilage on posteroanterior projections and this data are publicly available provided by the Boston University Clinical Epidemiology Research and Training Unit and the OAI Coordination Center. Thus, we identified this subset of participants (Fig. 1).

### 2.3 Hand radiographs and IAM

Posteroanterior radiographs of the dominant or left hand (in ambidextrous participants) were obtained. However, data on the presence of radiographic hand IAM is not publicly available from the dataset and was therefore assessed in the triangular fibrocartilage complexes by R.K. (a fellowship-trained musculoskeletal radiologist with 7 years of experience) (Fig. 1).

Presence of any IAM was defined as IAM presence in the assessed hand, ipsilateral or contralateral knee radiographs.

### 2.4 Statistical analysis

We compared baseline characteristics of participants with and without any IAM presence. Non-normality was found for age and Physical Activity Scale for the Elderly (PASE) scores using the Shapiro-Wilk test. Thus, Wilcoxon Rank-Sum tests for quantitative and Chi-square or Fisher's exact tests for categorical variables were employed for comparison. We explored the relationship between IAM and age using a logistic regression model adjusted for possible confounders.

**Fig. 2:**
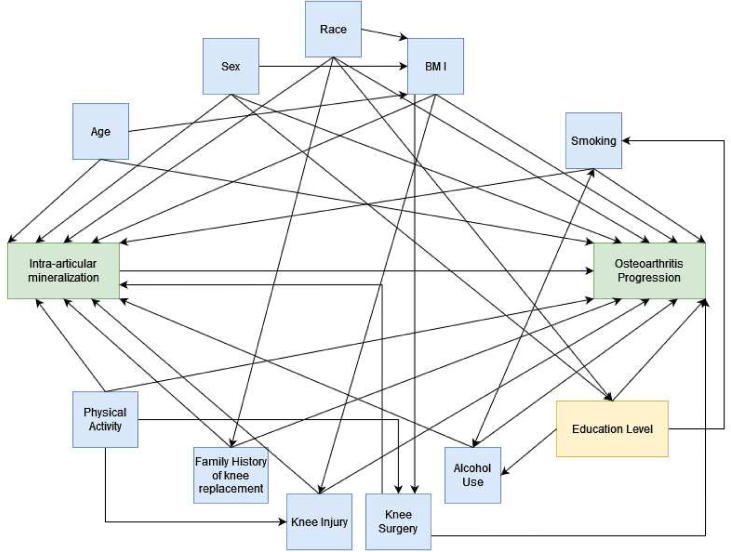
Direct Acyclic Graph for the relationship between intra-articular mineralization and osteoarthritis progression.

Relevant confounders were identified using a direct acyclic graph are as follows: age, sex, race (White/non-White), Body Mass Index (BMI), education level, weekly alcohol consumption, smoking status, physical activity (PASE Score), family history of knee replacement, history of injury in either knee that resulted in difficult ambulation for ≥1 week, and history of knee surgery (Fig. 2).

The primary outcome measure was the time to whole-grade OARSI-JSN progression of any compartment in any knee joint with baseline radiographic OA. In case both osteoarthritic knees at baseline showed radiographic progression during the follow up time, the earliest time to progression was used.

Using a Cox proportional-hazards regression model, we compared the risk of radiographic progression between participants with and without IAM presence, adjusted for confounders. Data were right-censored at time of last knee radiograph acquisition. Hazard ratios (HRs) and 95% confidence intervals (CIs) for IAM are reported. Based on the baseline characteristics of the participants, we hypothesized that IAM may contribute to OA progression in younger participants. Therefore, we conducted stratified analyses for participants aged ≥60 and ​< ​60.

Additionally, we analyzed the estimated effect of IAM on WOMAC total scores in eligible knees over a 9-year follow-up period using longitudinal multilevel regression models on a per knee basis which were clustered for participant ID to account for within individual variability when both knees of the same patient were eligible and also were adjusted for confounders. Stratified analysis was conducted for the knees of participants aged ≥60 and ​< ​60.

Analyses were conducted using the open-source R software version 4.2.0 (haven, survival, dplyr, survminer, nlme, and ggplot2 packages; R Foundation for Statistical Computing).

## 3 Results

### 3.1 Baseline characteristics

Our sample of 2010 participants (178 participants with any IAM and 1832 participants without IAM) had a median age of 64 years and 55% of participants were women (Table 1). Participants with any IAM (median: 69 years, interquartile range (IQR): 62–74) were older than participants without IAM (median: 63 years, IQR: 56–70) (p-value<0.001). In addition to age, we found differences in race (85% White participants with any IAM vs. 77% White participants without IAM, p-value: 0.018) and BMI (50% above median BMI participants with any IAM vs. 60% above median BMI participants without IAM, p-value: 0.003) between the two groups.

**Table 1:** Baseline characteristics of participants based on the presence of radiographic knee or hand intra-articular mineralization (IAM).

Characteristic	Overall, N ​= ​2,010^a^	Knee IAM, N ​= ​166^a^	Hand IAM, N ​= ​46^a^	Any (Knee or Hand) IAM, N ​= ​178^a^	No IAM, N ​= ​1,832^a^	*p*-value^b^
**Age (years)**	64.0 (56.0, 71.0)	69.0 (62.0, 74.0)	74.0 (69.0, 76.0)	69.0 (62.0, 74.0)	63.0 (56.0, 70.0)	**<0.001**
**Sex**						0.050
Male	904/2010 (45%)	89/166 (54%)	17/46 (37%)	93/178 (52%)	811/1832 (44%)	
Female	1106/2010 (55%)	77/166 (46%)	29/46 (63%)	85/178 (48%)	1021/1832 (56%)	
**Race**						**0.018**
White	1557/2009 (78%)	139/166 (84%)	42/46 (91%)	151/178 (85%)	1406/1831 (77%)	
Non-White	452/2009 (22%)	27/166 (16%)	4/46 (8.7%)	27/178 (15%)	425/1831 (23%)	
**BMI**						**0.003**
1st quartile: (16.9,25.1]	341/2006 (17%)	36/165 (22%)	13/46 (28%)	42/177 (24%)	299/1829 (16%)	
2nd quartile: (25.1,28.2]	478/2006 (24%)	45/165 (27%)	7/46 (15%)	47/177 (27%)	431/1829 (24%)	
3rd quartile: (28.2,31.7]	564/2006 (28%)	51/165 (31%)	15/46 (33%)	53/177 (30%)	511/1829 (28%)	
4th quartile: (31.7,48.7]	623/2006 (31%)	33/165 (20%)	11/46 (24%)	35/177 (20%)	588/1829 (32%)	
**Education Level**						0.2
High school or less	359/1996 (18%)	37/165 (22%)	11/45 (24%)	40/177 (23%)	319/1819 (18%)	
Undergraduate degree/undergraduate education	910/1996 (46%)	71/165 (43%)	22/45 (49%)	77/177 (44%)	833/1819 (46%)	
Graduate degree/graduate education	727/1996 (36%)	57/165 (35%)	12/45 (27%)	60/177 (34%)	667/1819 (37%)	
**Weekly Alcohol Consumption**						>0.9
No units	398/1993 (20%)	33/164 (20%)	12/45 (27%)	35/176 (20%)	363/1817 (20%)	
<1 unit	737/1993 (37%)	54/164 (33%)	16/45 (36%)	60/176 (34%)	677/1817 (37%)	
1–3 units	274/1993 (14%)	28/164 (17%)	2/45 (4.4%)	28/176 (16%)	246/1817 (14%)	
4–7 units	289/1993 (15%)	22/164 (13%)	7/45 (16%)	24/176 (14%)	265/1817 (15%)	
8–14 units	187/1993 (9.4%)	16/164 (9.8%)	4/45 (8.9%)	18/176 (10%)	169/1817 (9.3%)	
15+ units	108/1993 (5.4%)	11/164 (6.7%)	4/45 (8.9%)	11/176 (6.2%)	97/1817 (5.3%)	
**Smoking Status**						0.057
Never smokers	1053/1985 (53%)	79/164 (48%)	17/45 (38%)	83/176 (47%)	970/1809 (54%)	
Current smokers	121/1985 (6.1%)	7/164 (4.3%)	1/45 (2.2%)	7/176 (4.0%)	114/1809 (6.3%)	
Former smokers	811/1985 (41%)	78/164 (48%)	27/45 (60) %	86/176 (49%)	725/1809 (40%)	
**Physical Activity Scale for the Elderly score**	144.0 (94.0, 206.0)	142.0 (86.0, 204.0)	120 (73.0, 169.0)	138.0 (87.0, 202.0)	145.0 (95.0, 206.0)	0.3
**Family History of Knee Replacement**	294/1986 (15%)	18/164 (11%)	5/46 (11%)	20/176 (11%)	274/1810 (15%)	0.2
**History of Ambulation-Altering Knee Injury**	954/1993 (48%)	89/165 (54%)	17/46 (37%)	92/177 (52%)	862/1816 (47%)	0.3
**History of Knee Surgery**	678/2009 (34%)	66/166 (39.7%)	14/46 (30%)	68/178 (38%)	610/1831 (33%)	0.2

a Median (Interquartile Range); n/N (%).

b Wilcoxon rank-sum Test; Pearson's Chi-squared test/Fisher's exact test; comparing No IAM vs. Any (hand or knee) IAM.

There was no difference between participants with and without IAM according to education, weekly alcohol consumption, smoking status, PASE scores, family history of knee replacement, and history of knee injury or surgery (p-values >0.05) (Table 1).

Exploratory evaluation of any IAM presence vs. age (categorized in 5-year intervals) was performed (Supplement-1). Using a logistic regression model adjusted for the above-mentioned confounders, we investigated the probability of any IAM vs. age (Odds Ratio: 1.06, 95% Confidence Interval (CI): 1.04–1.08) (Supplement-2).

### 3.2 OA-related radiographic progression

Twenty-six percent of participants with any IAM and 28% of participants without IAM developed radiographic whole-grade OARSI-JSN progression in eligible knees over the 8-year period. The risk of progression was not associated with baseline any IAM presence (HR: 1.00, 95%CI: 0.73–1.37) (Table 2).

**Table 2:** Results of cox proportional hazards models and longitudinal multilevel models investigating radiographic progression and clinical progression, respectively.

Sample Characteristic	Participants of all ages, N ​= ​2010			Participants aged ≥60, N ​= ​1270			Participants aged <60, N ​= ​740		
	HR	95% CI	p-value	HR	95% CI	p-value	HR	95% CI	p-value
**Hazard Ratios of Intra-articular mineralization (IAM) of Cox Proportional-Hazards Models for radiographic osteoarthritis progression in participants with age-based subgroup analysis**									
Any (Knee or Hand) IAM	1.00	0.73–1.37	0.99	0.89	0.62–1.29	0.54	**1.90**	**1.01–3.60**	**0.04**
Knee IAM	0.94	0.67–1.31	0.71	0.84	0.57–1.24	0.37	1.72	0.89–3.34	0.11
Hand IAM	1.59	0.97–2.60	0.07	1.40	0.81–2.42	0.23	**10.37**	**3.03–35.46**	**<0.001**

Sample Characteristic	Participants of all ages, N = 2010			Participants aged ≥60, N = 1270			Participants aged < 60, N = 740		
	Estimate	95% CI	p-value	Estimate	95% CI	p-value	Estimate	95% CI	p-value
**Estimated change in Total WOMAC score due to presence of Intra-articular mineralization (IAM) with age-based subgroup analysis using longitudinal multilevel models**									
Any (Knee or Hand) IAM	1.24	−0.38–2.86	0.13	1.55	−0.19–3.29	0.08	1.20	−2.72–5.12	0.55
Knee IAM	1.00	−0.67–2.68	0.24	1.39	−0.41–3.18	0.13	0.68	−3.31–4.68	0.74
Hand IAM	**3.63**	**0.64–6.61**	**0.02**	**3.20**	**0.11–6.29**	**0.04**	7.60	−1.55–16.75	0.10

All Cox Proportional-Hazards Models and longitudinal multilevel models were adjusted for age, sex, race, BMI, education level, weekly alcohol consumption, smoking status, Physical Activity Scale for the Elderly score, family history of knee replacement, history of knee injury resulting in difficult ambulation ≥1 week, and history of knee surgery.

In a stratified analysis of the participants aged <60, 35% of participants with any IAM and 27% without IAM developed whole-grade OARSI-JSN progression in either eligible knee. (p-value: 0.54) Risk of radiographic progression was associated with baseline any IAM presence (HR: 1.90, 95%CI: 1.01–3.60). No such association was found in participants aged ≥60 (HR: 0.89, 95%CI: 0.62–1.29) (Table 2).

Subgroup analyses were conducted on participants with vs. without knee IAM. Risk of whole-grade OARSI-JSN progression was not associated with knee IAM presence in all patients (HR: 0.94, 95%CI: 0.67–1.31), those aged ≥60 (HR: 0.84, 95%CI: 0.57–1.24), or those aged <60 (HR: 1.72, 95%CI: 0.89–3.34) (Table 2).

Presence of hand IAM was associated with radiographic progression of OA in participants aged <60 (HR: 10.37, 95%CI: 3.03–35.46) (Table 2).

All reported models met proportional-hazards assumptions.

### 3.3 OA-related symptom progression

No differences were observed in WOMAC scores between participants with vs. those without any IAM (Estimated difference:1.24, p-value: 0.13). Additionally, stratified analyses conducted for participants aged ≥60 (p-value: 0.08) and <60 (p-value: 0.55) years old showed no differences in WOMAC scores in eligible knees (Table 2).

Subgroup analyses were conducted on participants with and without knee IAM. No differences were observed in WOMAC scores of eligible knees between participants with vs. those without knee IAM in all subgroups (p-values >0.05) (Table 2).

Presence of hand IAM in all participants and participants aged ≥60 was associated with symptomatic progression of OA (Estimated difference:3.63, 3.20, p-value: 0.02, 0.04 respectively) (Table 2).

## 4 Discussion

In this study, 8.9% of OAI participants with baseline knee radiographic OA had radiographically detectable IAM. Participants with IAM were older, male, white, and of a lower BMI, consistent with the existing literature [13]. IAM is a common radiographic finding coexisting with knee OA, though the nature of its relationship with OA and OA progression is controversial. IAM has been proposed as a marker for chondrocyte apoptosis, local inflammation, and biomechanical stress [6].

We demonstrated that radiographically detectable IAM is not associated with an increased risk of radiographic OA progression for the entire sample. This is consistent with previous explorations of the relationship between baseline knee IAM and radiographic OA progression using longitudinal MRIs [1] and plain radiographs [5]. Neogi et al. hypothesized that IAM may be a marker for metabolically active chondrocytes as inorganic phosphates are produced as markers of chondrocyte hypertrophic response in addition to as substrates for calcium-containing crystals [1]. However, the mean age of participants in previous studies was high; therefore, their conclusions may not apply to younger (<60 years old) populations.

Following the stratification according to age, any IAM was associated with radiographic OA progression in participants <60 years old but not in those ≥60 years. This suggests a predominant role of early IAM in OA pathogenesis of younger patients. In other areas of the body such as the brain and costal cartilages, calcification at younger ages is known to be secondary to underlying disorders [7,10]. Therefore, it is plausible that in younger individuals, IAM is a result of an underlying pathology that could negatively impact OA outcomes, whereas IAM in older individuals may predominantly occur as a manifestation of physiologic aging. However, our findings revealed that the presence of knee IAM was not predictive of radiographic OA progression in either age group which might have been due to lower number of participants in comparison to those with any IAM.

There is evidence to suggest that IAM is a systemic process that involves multiple joints [14], and the most common sites for IAM are the knee and wrist [11]. Recent reports have shown advantages of CT in the detection and localization of IAM compared to conventional radiography [2]. However, due to the convenient use of radiographs in clinical practice, we chose to test our hypothesis using plain radiographs.

Our study has several limitations. Low sensitivity for detection and localization of IAM using plain radiographs compared to CT [11,12] may negatively affect the optimal assessment of the predictive value of IAM in OA-related outcomes. However, radiographs are a convenient tool for assessments of OA and IAM and therefore, remain a key part of clinical practice [11,15]. The OAI dataset contains data for participants with OA or at risk of development of OA. Hence, our results are not readily generalizable, though they may be reflective of an at-risk population. Third, radiographs were assessed annually, which may introduce bias to survival analysis due to interval censoring. The lack of publicly available robust MRI data on longitudinal OA-related structural damage occurrence within the OAI dataset, can be a subject for future investigations. Our study was unable to distinguish between IAM secondary to underlying metabolic derangements due to a lack of available measurements of serum calcium and other relevant markers. Moreover, since data on meniscal or ligamentous calcification is not available within the OAI dataset, our findings are limited to chondrocalcinosis. Finally, our study included a relatively small sample size of patients with IAM data available in OAI. However, it is the largest cohort to conduct a longitudinal observational study in OA patients.

In conclusion, our report suggests that IAM presence does not influence overall risk of radiographic and symptomatic knee OA progression. However, following stratification by age, participants <60 years old showed an association between any IAM and increased risk of radiographic knee OA progression. Future studies are warranted to investigate the distinct role of IAM in OA pathogenesis among young patients.

## Credit author Statement

**Hamza Ahmed Ibad:** Conceptualization, Methodology, Formal Analysis, Visualization, Writing- Original Draft. **Robert M. Kwee:** Investigation, Data Curation, Writing – Review and Editing, Visualization. **Elena Ghotbi:** Formal Analysis, Validation, Writing – Review and Editing. **Frank W. Roemer:** Writing – Review and Editing. **Ali Guermazi:** Writing – Review and Editing. **Shadpour Demehri:** Conceptualization, Methodology, Writing – Original Draft, Writing – Review and Editing.

## Conflicts of interest and source of funding

Ali Guermazi is a shareholder of BICL. He is a consultant to Pfizer, TissueGene, Regeneron, Novartis, AstraZeneca, and Merck-Serono. Frank W Roemer is a shareholder of BICL. He is a consultant to Grünenthal GmbH and Calibr (past 36 months) None of the remaining authors have any conflicting personal or financial relationships that could have influenced the results of this study. This research was supported by the NIH National Institute of Aging (NIA) under Award Number P01AG066603 and NIH National Institute of Arthritis and Musculoskeletal and Skin Diseases (NIAMS) under Award Number R01AR079620-01.

## Funding

This research was supported by the NIH National Institute on Aging (NIA) under Award Number P01AG066603 and NIH National Institute of Arthritis and Musculoskeletal and Skin Diseases (NIAMS) under Award Number R01AR079620-01.

## Contributors

All authors participated in the study design, interpretation of results, and drafting of the manuscript or critically revising it for relevant intellectual content.

## Participant consent

Subjects have given informed consent before participation in the Osteoarthritis Initiative (OAI) project.

## Ethics approval

The medical ethics review boards of the University of California, San Francisco (Approval Number: 10–00532) and the four clinical centers of osteoarthritis initiative project recognized the project as Health Insurance Portability and Accountability Act (HIPAA)-compliant.

## Data sharing statement

Subjects' de-identified clinical and demographic information is publicly available at the osteoarthritis initiative project data repository at https://oai.nih.gov. The R codes used in this work are available from the corresponding author upon reasonable requests.

## Declaration of competing interest

Ali Guermazi is a shareholder of BICL. He is a consultant to Pfizer, TissueGene, Regeneron, Novartis, AstraZeneca, and Merck-Serono. Frank W Roemer is a shareholder of BICL. He is a consultant to Grünenthal GmbH and Calibr (past 36 months) None of the remaining authors have any conflicting personal or financial relationships that could have influenced the results of this study.
