# Supplementary figures and images for: Radiographically detectable intra-articular mineralization: Predictor of knee osteoarthritis outcomes or only an indicator of aging? A brief report from the osteoarthritis initiative
Source: Osteoarthr Cartil Open. 2023 Feb 23;5(2):100348. doi: 10.1016/j.ocarto.2023.100348 (PMC10009540; doi:10.1016/j.ocarto.2023.100348)

## Slide 1
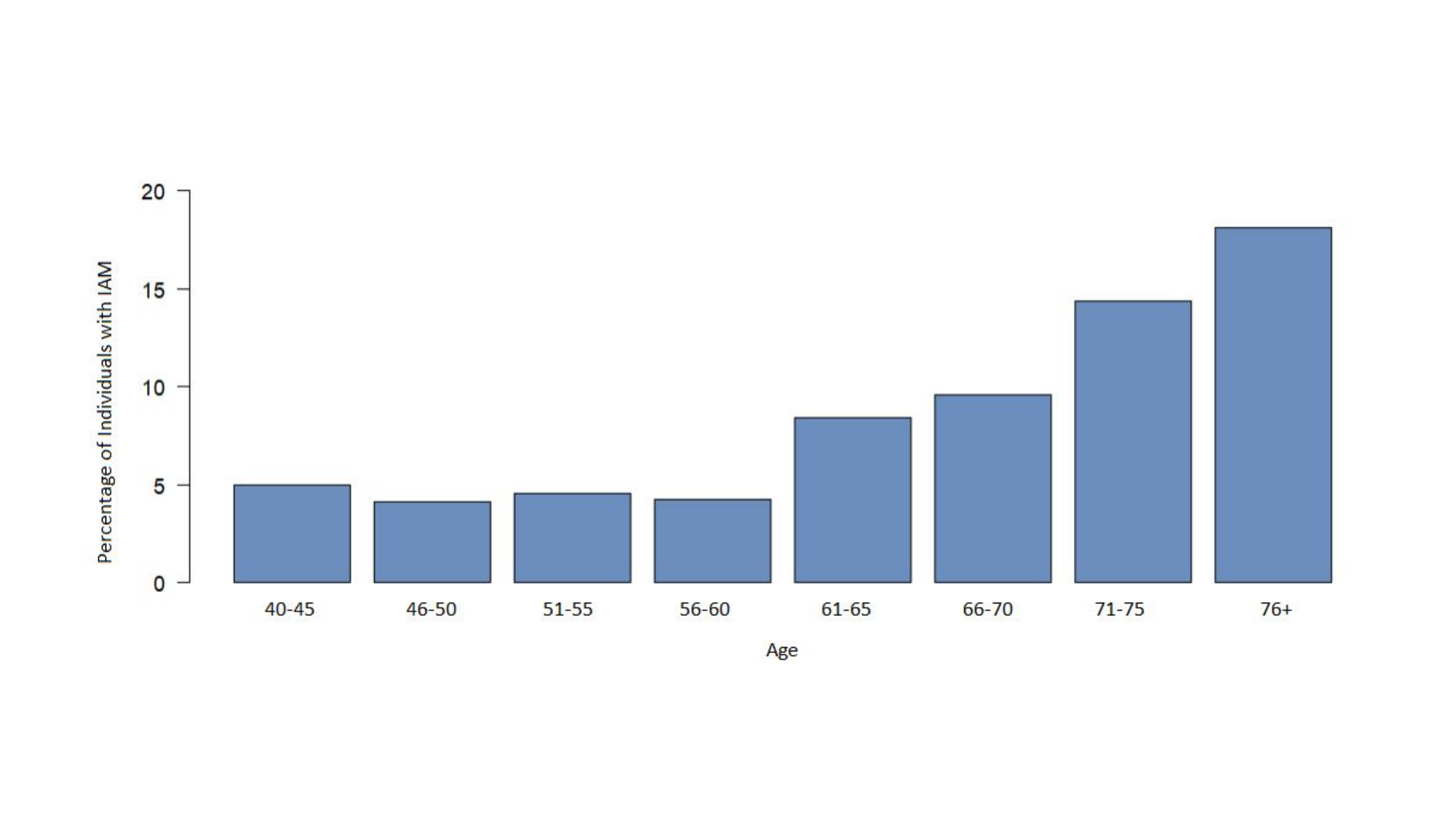

Supplement: Multimedia component 1 [file mmc1.pptx]

## Slide 1
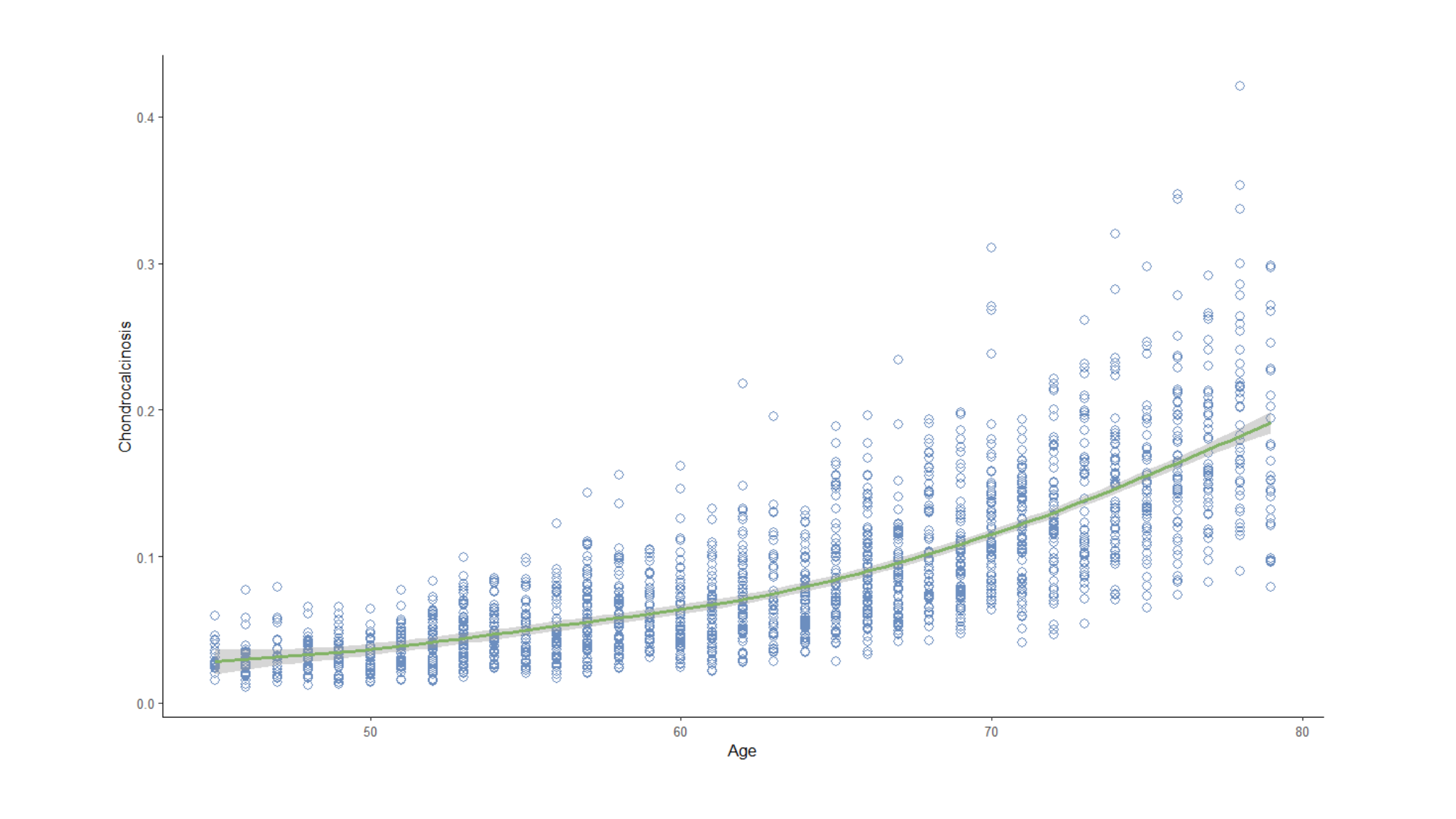

Supplement: Multimedia component 2 [file mmc2.pptx]
