# Supplementary material for: Radiographically detectable intra-articular mineralization: Predictor of knee osteoarthritis outcomes or only an indicator of aging? A brief report from the osteoarthritis initiative
Source: Osteoarthr Cartil Open. 2023 Feb 23;5(2):100348. doi: 10.1016/j.ocarto.2023.100348 (PMC10009540; doi:10.1016/j.ocarto.2023.100348)
Supplement: Multimedia component 3 [file mmc3.docx]

| **Supplement 2: Baseline characteristics of participants based on the presence of Knee Intra-articular Mineralization (IAM)** | | | |
| --- | --- | --- | --- |
| **Characteristic** | No Knee IAM, N = 1,844*^1^* | Knee IAM, N = 166*^1^* | p-value*^2^* |
| **Age (years)** | 63.0 (56.0, 70.0) | 69.0 (62.0, 74.0) | **<0.001** |
| **Sex** |  |  | **0.024** |
| Male | 815 / 1,844 (44%) | 89 / 166 (54%) |  |
| Female | 1,029 / 1,844 (56%) | 77 / 166 (46%) |  |
| **Race** |  |  | 0.056 |
| White | 1,418 / 1,843 (77%) | 139 / 166 (84%) |  |
| Non-White | 425 / 1,843 (23%) | 27 / 166 (16%) |  |
| **BMI** |  |  | **0.012** |
| 1^st^ quartile: (16.9,25.1] | 305 / 1,841 (17%) | 36 / 165 (22%) |  |
| 2^nd^ quartile: (25.1,28.2] | 433 / 1,841 (24%) | 45 / 165 (27%) |  |
| 3^rd^ quartile: (28.2,31.7] | 513 / 1,841 (28%) | 51 / 165 (31%) |  |
| 4^th^ quartile: (31.7,48.7] | 590 / 1,841 (32%) | 33 / 165 (20%) |  |
| **Education Level** |  |  | 0.3 |
| High school or less | 322 / 1,831 (18%) | 37 / 165 (22%) |  |
| Undergraduate degree/undergraduate education | 839 / 1,831 (46%) | 71 / 165 (43%) |  |
| Graduate degree/graduate education | 670 / 1,831 (37%) | 57 / 165 (35%) |  |
| **Weekly Alcohol Consumption** |  |  | 0.7 |
| No units | 365 / 1,829 (20%) | 33 / 164 (20%) |  |
| <1 unit | 683 / 1,829 (37%) | 54 / 164 (33%) |  |
| 1-3 units | 246 / 1,829 (13%) | 28 / 164 (17%) |  |
| 4-7 units | 267 / 1,829 (15%) | 22 / 164 (13%) |  |
| 8-14 units | 171 / 1,829 (9.3%) | 16 / 164 (9.8%) |  |
| 15+ units | 97 / 1,829 (5.3%) | 11 / 164 (6.7%) |  |
| **Smoking Status** |  |  | 0.2 |
| Never smokers | 974 / 1,821 (53%) | 79 / 164 (48%) |  |
| Current smokers | 114 / 1,821 (6.3%) | 7 / 164 (4.3%) |  |
| Former smokers | 733 / 1,821 (40%) | 78 / 164 (48%) |  |
| **Physical Activity Scale for the Elderly score** | 145.0 (95.0, 206.0) | 142.0 (86.0, 204.0) | 0.6 |
| **Family history of Knee Replacement** | 276 / 1,822 (15%) | 18 / 164 (11%) | 0.2 |
| **History of an ambulation-altering knee injury** | 865 / 1,828 (47%) | 89 / 165 (54%) | 0.12 |
| *^1^*Median (Interquartile Range); n / N (%) *^2^*Wilcoxon rank-sum Test; Pearson's Chi-squared test/Fisher’s exact test | | | |
